# {[2-Methyl-2-(phen­oxy­meth­yl)propane-1,3-di­yl]bis­(­oxy)}di­benzene

**DOI:** 10.1107/S1600536814007594

**Published:** 2014-04-12

**Authors:** Ziad Moussa, Harbi Tomah Al-Masri, Amjed Shraim, Mohammed Fettouhi

**Affiliations:** a Department of Chemistry, Faculty of Science, Taibah University, PO Box 30002, Almadinah Almunawarrah, Saudi Arabia; b Department of Chemistry and Earth Sciences, College of Arts and Sciences, Qatar University, PO Box 2713, Doha, State of Qatar; c Department of Chemistry, King Fahd University of Petroleum and Minerals, 31261 Dhahran, Saudi Arabia

## Abstract

The title compound, C_23_H_24_O_3_, was obtained in a one-step (60% yield) synthesis from 1,1,1-tris(hydroxymethyl)ethane. It features a tripodal ligand capable of complexing metal centres. One of the three conformations involving the methyl group, the central C—C bond and the phenoxy substituents is antiperiplanar while the two others are synclinal [the corresponding C—C—C—O torsion angles are −174.6 (1), −53.2 (2) and −47.3 (2)°]. In the crystal, C—H⋯O inter­actions link the molecules into [010] chains.

## Related literature   

For details of the synthesis, see: Viguier *et al.* (2001 ▶); Alajarín *et al.* (2007 ▶); Beaufort *et al.* (2007 ▶). For a related structure, see: Laliberté *et al.* (2003 ▶).
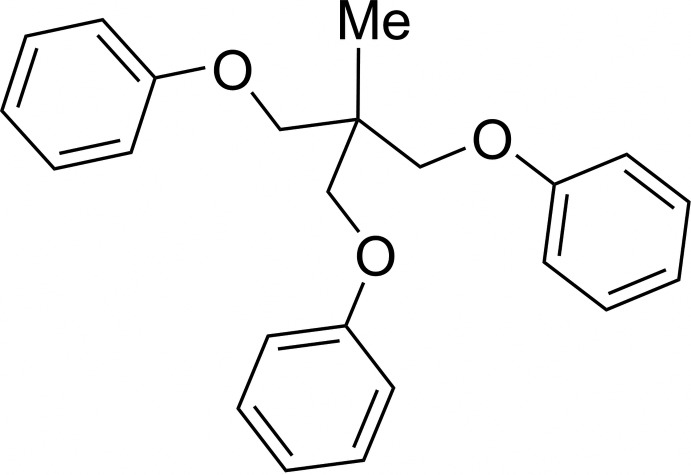


## Experimental   

### 

#### Crystal data  

C_23_H_24_O_3_
*M* *_r_* = 348.42
Monoclinic, 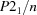

*a* = 13.5755 (15) Å
*b* = 6.2829 (7) Å
*c* = 22.514 (3) Å
β = 91.033 (2)°
*V* = 1920.0 (4) Å^3^
*Z* = 4
Mo *K*α radiation
μ = 0.08 mm^−1^
*T* = 295 K
0.41 × 0.32 × 0.11 mm

#### Data collection  

Bruker SMART APEX area-detector diffractometer
Absorption correction: multi-scan (*SADABS*; Sheldrick, 1996 ▶) *T*_min_ = 0.969, *T*_max_ = 0.991
16758 measured reflections
4770 independent reflections
2614 reflections with *I* > 2σ(*I*)
*R*_int_ = 0.045

#### Refinement  

*R*[*F* ^2^ > 2σ(*F* ^2^)] = 0.050
*wR*(*F* ^2^) = 0.124
*S* = 1.00
4770 reflections
236 parameters
H-atom parameters constrained
Δρ_max_ = 0.16 e Å^−3^
Δρ_min_ = −0.14 e Å^−3^

### 

Data collection: *SMART* (Bruker, 2001 ▶); cell refinement: *SAINT* (Bruker, 2001 ▶); data reduction: *SAINT*; program(s) used to solve structure: *SHELXS97* (Sheldrick, 2008 ▶); program(s) used to refine structure: *SHELXL97* (Sheldrick, 2008 ▶); molecular graphics: *ORTEP-3 for Windows* (Farrugia, 2012 ▶); software used to prepare material for publication: *SHELXL97*.

## Supplementary Material

Crystal structure: contains datablock(s) I, New_Global_Publ_Block. DOI: 10.1107/S1600536814007594/bt6973sup1.cif

Structure factors: contains datablock(s) I. DOI: 10.1107/S1600536814007594/bt6973Isup2.hkl

Supporting information file. DOI: 10.1107/S1600536814007594/bt6973Isup3.cml

CCDC reference: 942596

Additional supporting information:  crystallographic information; 3D view; checkCIF report

## Figures and Tables

**Table 1:** Hydrogen-bond geometry (Å, °)

*D*—H⋯*A*	*D*—H	H⋯*A*	*D*⋯*A*	*D*—H⋯*A*
C5—H5⋯O2^i^	0.93	2.59	3.5081 (19)	170

Symmetry code: (i) 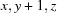.

**Fig. 1.**
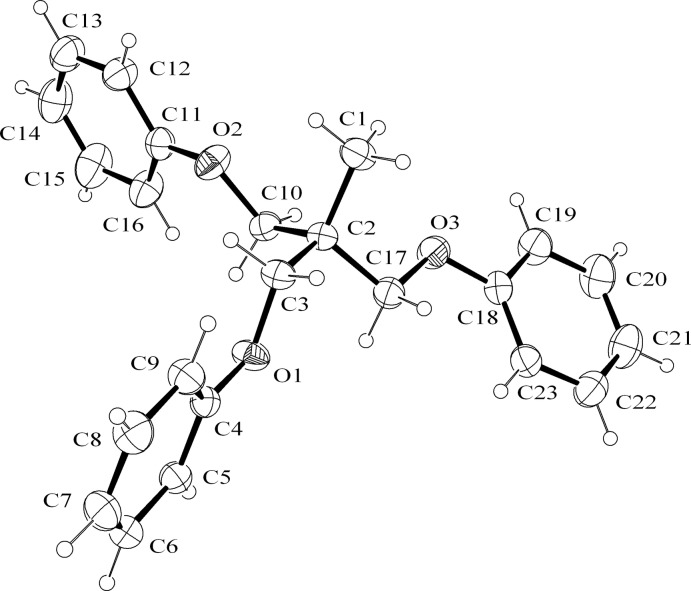
The molecular structure of the title compound. Displacement ellipsoids are drawn at the 50% probability level and H atoms are shown as small spheres of arbitrary radius.

## supplementary crystallographic information

### 1. Comment

α,α,α*-*tris(hydroxymethyl)ethane has been widely used in the design of polypodal ligands (Viguier *et al.*, 2001; Alajarín *et al.*, 2007; Beaufort *et al.*, 2007) capable of forming stable complexes with transition metals [Cu(I), Cu(II), Ni(II), Pd(II), Y(III)] and a variety of lanthanide(III) cations (La^3+^, Sm^3+^, Eu^3+^, Gd^3+^, Tb^3+^, Dy^3+^). The main step in the preparation of such compounds involves nucleophilic displacement of the hydroxyl group with various nucleophiles. The hydroxyl group is initially converted to a tosylate (Beaufort *et al.*, 2007) or a halogen (Alajarín *et al.*, 2007) before the substitution step is carried out. The title compound provides a related tripodal ligand that can be readily synthesized in a single step and in good yield. In the course of investigating its use as a tripodal ligand in transition metal complexation reactions, we examined its structure to determine the preferred conformation, identify the principal intermolecular interactions, and extract detailed geometric information.

Initial attempts to prepare the title compound by reacting phenol or sodium phenoxide with α,α,α-tris[(4-tolylsulfonyl)methyl]ethane or α,α,α*-*tris(chloromethyl)ethane were unsuccessful due to the poor nucleophilic character of phenol and its alkali metal salts. However, converting α,α,α*-*tris(hydroxymethyl)ethane to the corresponding trifluoromethanesulfonate derivative gave a more effective substrate with a much superior leaving group ability. Thus, the latter derivative reacted with sodium phenoxide under very mild conditions to afford the title compound in 60% isolated yield.

The X-ray structure determination of the tripodal *O,O,O*-ligand shows the central C2-atom to be bonded to a methyl groups and three phenoxymethyl groups. The geometry around the central C-atom could be described as a slightly distorted tetrahedron because the bond angles deviate from the ideal value of 109.47°. The C(3)-C(2)-C(10) [111.04 (13)°] and C(1)-C(2)-C(17) [110.26 (13)°], and C(1)-C(2)-C(10) [111.15 (13)°] angles are wide, and the other three angles are narrow. The three phenoxymethyl arms are tilted away from the C-center due to steric interactions. One of the three conformations involving the methyl group, the central C—C bond and each one of the three phenoxy substituents is antiperiplanar while the two others are synclinal. The corresponding torsion angles are C1—C2—C3—O1: -174.6 (1)°, C1—C2—C17—O3: -53.2 (2)° and C1—C2—C10—O2: -47.3 (2)° respectively. The bond angles and bond distances are in good agreement with those reported for the only one reported analog namely 1,3-diphenoxy-2,2-bis(phenoxymethyl)propane (Laliberté *et al.*, 2003).

The only remarkable short intermolecular contact is a C-H···O interaction.

### 2. Experimental

Preparation of (2-methyl-2-(phenoxymethyl)propane-1,3-diyl)bis(oxy)dibenzene

1,1,1*-*tris*(*hydroxymethyl*)*ethane (600 mg, 5 mmol) was dissolved in pyridine (10 ml) and cooled to 273K in an ice/water bath. The colorless solution was treated dropwise over ten minutes with trifluoromethanesulfonic anhydride (4.34 g, 2.6 ml, 15.4 mmol) to give a deep dark red homogeneous solution and stirring was continued for another 50 minutes. Simultaneously and in a separate flask, NaH (1.98 g, 60%, 50 mmol) was washed with hexanes and suspended in THF (30 ml) at 273K. Phenol (4.23 g, 45 mmol) was added in portions to the stirred suspension over 1 h. The trifluoromethanesulfonate solution was then slowly added to the sodium phenoxide solution at 273K to give a light reddish yellow color. The ice bath was removed and the mixture was subsequently stirred overnight at room temperature. The mixture was diluted with diethyl ether (50 ml) and the ether layer was washed with 5% HCl solution (3 *x* 20 ml), 1 N solution of NaOH (3 *x* 20 ml), saturated NaCl solution (3 *x* 20 ml), dried (Na_2_SO_4_) and concentrated *in vacuo*. ^1^H NMR analysis of the crude indicated that it consisted of a 2:1 mixture of the product and corresponding disubstituted analogue. The residue was initially chromatographed (elution with 90% hexanes-ethyl acetate) to provide an unseparated mixture of the aforementioned products. Further chromatographic separation with hexanes and re-crystallization (hexanes) afforded 1.04 g (60%) of the tripodal ligand as a colorless crystalline solid: ^1^H NMR (CDCl_3_, 400 MHz) δ 7.30–7.20 (m, 6H, Ar—H), 6.97–6.88 (m, 9H, Ar—H), 4.09 (s, 6H, OCH_2_), 1.33 (s, 3H, CH_3_); ^13^C NMR (CDCl_3_, 100 MHz) δ 159.1 (C), 129.4 (CH), 120.8 (CH), 114.6 (CH), 70.0 (CH_2_), 40.4 (C), 17.3 (CH_3_).

### 3. Refinement

All the H atoms were positioned geometrically (C—H = 0.93–0.97 Å) and refined as riding with *U*iso(H) = 1.2*U*eq(C) or 1.5*U*eq(methyl C).

### Crystal data

**Table:**

C_23_H_24_O_3_	*F*(000) = 744
*M**_r_* = 348.42	*D*_x_ = 1.205 Mg m^−^^3^
Monoclinic, *P*2_1_/*n*	Melting point: 340 K
Hall symbol: -P 2yn	Mo *K*α radiation, λ = 0.71073 Å
*a* = 13.5755 (15) Å	Cell parameters from 16758 reflections
*b* = 6.2829 (7) Å	θ = 1.7–28.3°
*c* = 22.514 (3) Å	µ = 0.08 mm^−^^1^
β = 91.033 (2)°	*T* = 295 K
*V* = 1920.0 (4) Å^3^	Block, colourless
*Z* = 4	0.41 × 0.32 × 0.11 mm

### Data collection

**Table:**

Bruker SMART APEX area-detector diffractometer	4770 independent reflections
Radiation source: normal-focus sealed tube	2614 reflections with *I* > 2σ(*I*)
Graphite monochromator	*R*_int_ = 0.045
ω scans	θ_max_ = 28.3°, θ_min_ = 1.7°
Absorption correction: multi-scan (*SADABS*; Sheldrick, 1996)	*h* = −18→18
*T*_min_ = 0.969, *T*_max_ = 0.991	*k* = −8→8
16758 measured reflections	*l* = −26→29

### Refinement

**Table:**

Refinement on *F*^2^	Primary atom site location: structure-invariant direct methods
Least-squares matrix: full	Secondary atom site location: difference Fourier map
*R*[*F*^2^ > 2σ(*F*^2^)] = 0.050	Hydrogen site location: inferred from neighbouring sites
*wR*(*F*^2^) = 0.124	H-atom parameters constrained
*S* = 1.00	*w* = 1/[σ^2^(*F*_o_^2^) + (0.0497*P*)^2^ + 0.108*P*] where *P* = (*F*_o_^2^ + 2*F*_c_^2^)/3
4770 reflections	(Δ/σ)_max_ < 0.001
236 parameters	Δρ_max_ = 0.16 e Å^−^^3^
0 restraints	Δρ_min_ = −0.14 e Å^−^^3^

### Special details

**Table:**

Geometry. All e.s.d.'s (except the e.s.d. in the dihedral angle between two l.s. planes) are estimated using the full covariance matrix. The cell e.s.d.'s are taken into account individually in the estimation of e.s.d.'s in distances, angles and torsion angles; correlations between e.s.d.'s in cell parameters are only used when they are defined by crystal symmetry. An approximate (isotropic) treatment of cell e.s.d.'s is used for estimating e.s.d.'s involving l.s. planes.
Refinement. Refinement of *F*^2^ against ALL reflections. The weighted *R*-factor *wR* and goodness of fit *S* are based on *F*^2^, conventional *R*-factors *R* are based on *F*, with *F* set to zero for negative *F*^2^. The threshold expression of *F*^2^ > σ(*F*^2^) is used only for calculating *R*-factors(gt) *etc*. and is not relevant to the choice of reflections for refinement. *R*-factors based on *F*^2^ are statistically about twice as large as those based on *F*, and *R*- factors based on ALL data will be even larger.

### Fractional atomic coordinates and isotropic or equivalent isotropic displacement parameters (Å^2^)

**Table:**

	*x*	*y*	*z*	*U*_iso_*/*U*_eq_	
O1	0.68030 (8)	0.63321 (17)	0.10973 (5)	0.0513 (3)	
O2	0.82639 (8)	0.16266 (19)	0.06970 (5)	0.0576 (3)	
O3	0.68819 (9)	0.4660 (2)	−0.06922 (5)	0.0603 (3)	
C1	0.64308 (13)	0.1538 (3)	0.01456 (8)	0.0575 (5)	
H1A	0.6750	0.1020	−0.0203	0.086*	
H1B	0.5742	0.1738	0.0060	0.086*	
H1C	0.6512	0.0524	0.0462	0.086*	
C2	0.68907 (11)	0.3661 (2)	0.03331 (7)	0.0431 (4)	
C3	0.64647 (12)	0.4268 (3)	0.09311 (7)	0.0469 (4)	
H3A	0.5751	0.4260	0.0904	0.056*	
H3B	0.6669	0.3239	0.1230	0.056*	
C4	0.65081 (11)	0.7116 (3)	0.16385 (6)	0.0423 (4)	
C5	0.69030 (11)	0.9064 (3)	0.17973 (7)	0.0470 (4)	
H5	0.7327	0.9762	0.1543	0.056*	
C6	0.66682 (13)	0.9972 (3)	0.23330 (7)	0.0564 (5)	
H6	0.6927	1.1293	0.2437	0.068*	
C7	0.60515 (14)	0.8929 (3)	0.27141 (8)	0.0643 (5)	
H7	0.5905	0.9526	0.3080	0.077*	
C8	0.56545 (14)	0.7011 (3)	0.25532 (8)	0.0627 (5)	
H8	0.5231	0.6322	0.2810	0.075*	
C9	0.58723 (12)	0.6073 (3)	0.20120 (7)	0.0524 (4)	
H9	0.5596	0.4773	0.1904	0.063*	
C10	0.80135 (11)	0.3501 (3)	0.03726 (7)	0.0470 (4)	
H10A	0.8284	0.3432	−0.0023	0.056*	
H10B	0.8284	0.4744	0.0573	0.056*	
C11	0.92434 (11)	0.1140 (3)	0.07759 (7)	0.0484 (4)	
C12	0.94461 (14)	−0.0863 (3)	0.10011 (8)	0.0616 (5)	
H12	0.8935	−0.1802	0.1079	0.074*	
C13	1.04092 (15)	−0.1463 (4)	0.11106 (9)	0.0717 (6)	
H13	1.0545	−0.2815	0.1259	0.086*	
C14	1.11664 (15)	−0.0090 (4)	0.10026 (9)	0.0795 (6)	
H14	1.1815	−0.0497	0.1079	0.095*	
C15	1.09607 (14)	0.1878 (4)	0.07819 (10)	0.0836 (7)	
H15	1.1475	0.2814	0.0710	0.100*	
C16	1.00011 (13)	0.2518 (3)	0.06624 (8)	0.0655 (5)	
H16	0.9872	0.3863	0.0507	0.079*	
C17	0.66205 (12)	0.5391 (3)	−0.01163 (7)	0.0498 (4)	
H17A	0.5919	0.5682	−0.0106	0.060*	
H17B	0.6973	0.6693	−0.0021	0.060*	
C18	0.66232 (11)	0.5878 (3)	−0.11761 (7)	0.0494 (4)	
C19	0.68929 (13)	0.5068 (3)	−0.17178 (8)	0.0652 (5)	
H19	0.7227	0.3778	−0.1737	0.078*	
C20	0.66669 (15)	0.6174 (4)	−0.22296 (9)	0.0786 (6)	
H20	0.6847	0.5621	−0.2595	0.094*	
C21	0.61777 (15)	0.8086 (4)	−0.22066 (9)	0.0769 (6)	
H21	0.6030	0.8831	−0.2554	0.092*	
C22	0.59107 (13)	0.8883 (3)	−0.16677 (9)	0.0664 (5)	
H22	0.5577	1.0174	−0.1651	0.080*	
C23	0.61290 (12)	0.7797 (3)	−0.11474 (8)	0.0543 (5)	
H23	0.5946	0.8352	−0.0783	0.065*	

### Atomic displacement parameters (Å^2^)

**Table:**

	*U*^11^	*U*^22^	*U*^33^	*U*^12^	*U*^13^	*U*^23^
O1	0.0664 (7)	0.0418 (7)	0.0462 (6)	−0.0108 (6)	0.0132 (5)	−0.0045 (5)
O2	0.0462 (6)	0.0552 (8)	0.0716 (8)	0.0021 (5)	0.0038 (5)	0.0187 (6)
O3	0.0792 (8)	0.0587 (8)	0.0431 (6)	0.0172 (6)	0.0000 (6)	−0.0039 (6)
C1	0.0607 (10)	0.0499 (11)	0.0618 (11)	−0.0042 (9)	−0.0006 (8)	−0.0106 (9)
C2	0.0465 (9)	0.0377 (9)	0.0450 (9)	0.0000 (7)	0.0016 (7)	−0.0034 (7)
C3	0.0514 (9)	0.0395 (10)	0.0500 (10)	−0.0053 (7)	0.0047 (7)	−0.0030 (7)
C4	0.0476 (9)	0.0421 (10)	0.0371 (8)	0.0025 (7)	0.0002 (7)	0.0013 (7)
C5	0.0520 (9)	0.0431 (10)	0.0459 (9)	−0.0017 (8)	0.0006 (7)	0.0026 (8)
C6	0.0676 (11)	0.0482 (11)	0.0531 (10)	0.0021 (9)	−0.0083 (9)	−0.0066 (9)
C7	0.0793 (13)	0.0702 (14)	0.0435 (10)	0.0072 (11)	0.0027 (9)	−0.0106 (10)
C8	0.0680 (11)	0.0719 (15)	0.0488 (10)	−0.0004 (10)	0.0141 (9)	0.0055 (10)
C9	0.0585 (10)	0.0504 (11)	0.0484 (10)	−0.0070 (8)	0.0057 (8)	0.0010 (8)
C10	0.0512 (9)	0.0416 (10)	0.0483 (9)	0.0002 (7)	0.0034 (7)	0.0037 (8)
C11	0.0469 (9)	0.0559 (12)	0.0425 (9)	0.0015 (8)	0.0022 (7)	0.0006 (8)
C12	0.0627 (11)	0.0554 (12)	0.0666 (12)	0.0047 (9)	−0.0001 (9)	0.0038 (10)
C13	0.0754 (14)	0.0700 (15)	0.0696 (13)	0.0224 (12)	−0.0040 (10)	0.0024 (11)
C14	0.0534 (12)	0.110 (2)	0.0745 (14)	0.0163 (12)	−0.0008 (10)	0.0097 (14)
C15	0.0505 (12)	0.108 (2)	0.0927 (16)	−0.0062 (12)	0.0015 (10)	0.0268 (14)
C16	0.0534 (11)	0.0731 (14)	0.0701 (12)	−0.0013 (10)	0.0019 (9)	0.0184 (11)
C17	0.0528 (9)	0.0512 (11)	0.0453 (9)	0.0064 (8)	0.0006 (7)	−0.0051 (8)
C18	0.0448 (9)	0.0575 (12)	0.0456 (9)	−0.0024 (8)	−0.0038 (7)	0.0010 (8)
C19	0.0677 (12)	0.0767 (15)	0.0513 (11)	0.0111 (10)	0.0068 (9)	−0.0016 (10)
C20	0.0789 (14)	0.109 (2)	0.0487 (11)	0.0070 (13)	0.0101 (10)	0.0062 (12)
C21	0.0679 (13)	0.1007 (19)	0.0622 (13)	0.0049 (12)	0.0022 (10)	0.0246 (12)
C22	0.0604 (11)	0.0630 (13)	0.0757 (14)	0.0007 (10)	−0.0049 (10)	0.0130 (11)
C23	0.0544 (10)	0.0543 (12)	0.0539 (10)	−0.0033 (9)	−0.0044 (8)	−0.0009 (9)

### Geometric parameters (Å, º)

**Table:**

O1—C4	1.3804 (17)	C10—H10A	0.9700
O1—C3	1.4238 (18)	C10—H10B	0.9700
O2—C11	1.3729 (19)	C11—C16	1.372 (2)
O2—C10	1.4240 (18)	C11—C12	1.382 (2)
O3—C18	1.3718 (19)	C12—C13	1.379 (3)
O3—C17	1.4263 (18)	C12—H12	0.9300
C1—C2	1.529 (2)	C13—C14	1.368 (3)
C1—H1A	0.9600	C13—H13	0.9300
C1—H1B	0.9600	C14—C15	1.359 (3)
C1—H1C	0.9600	C14—H14	0.9300
C2—C3	1.523 (2)	C15—C16	1.385 (3)
C2—C17	1.525 (2)	C15—H15	0.9300
C2—C10	1.529 (2)	C16—H16	0.9300
C3—H3A	0.9700	C17—H17A	0.9700
C3—H3B	0.9700	C17—H17B	0.9700
C4—C5	1.380 (2)	C18—C19	1.377 (2)
C4—C9	1.381 (2)	C18—C23	1.382 (2)
C5—C6	1.377 (2)	C19—C20	1.375 (3)
C5—H5	0.9300	C19—H19	0.9300
C6—C7	1.376 (2)	C20—C21	1.374 (3)
C6—H6	0.9300	C20—H20	0.9300
C7—C8	1.366 (3)	C21—C22	1.367 (3)
C7—H7	0.9300	C21—H21	0.9300
C8—C9	1.390 (2)	C22—C23	1.383 (2)
C8—H8	0.9300	C22—H22	0.9300
C9—H9	0.9300	C23—H23	0.9300
			
C4—O1—C3	117.37 (11)	H10A—C10—H10B	108.4
C11—O2—C10	118.19 (12)	C16—C11—O2	124.26 (16)
C18—O3—C17	118.56 (13)	C16—C11—C12	119.85 (16)
C2—C1—H1A	109.5	O2—C11—C12	115.86 (15)
C2—C1—H1B	109.5	C13—C12—C11	119.77 (18)
H1A—C1—H1B	109.5	C13—C12—H12	120.1
C2—C1—H1C	109.5	C11—C12—H12	120.1
H1A—C1—H1C	109.5	C14—C13—C12	120.6 (2)
H1B—C1—H1C	109.5	C14—C13—H13	119.7
C3—C2—C17	108.50 (13)	C12—C13—H13	119.7
C3—C2—C1	107.62 (13)	C15—C14—C13	119.26 (19)
C17—C2—C1	110.26 (13)	C15—C14—H14	120.4
C3—C2—C10	111.04 (13)	C13—C14—H14	120.4
C17—C2—C10	108.24 (12)	C14—C15—C16	121.4 (2)
C1—C2—C10	111.15 (13)	C14—C15—H15	119.3
O1—C3—C2	109.54 (12)	C16—C15—H15	119.3
O1—C3—H3A	109.8	C11—C16—C15	119.08 (19)
C2—C3—H3A	109.8	C11—C16—H16	120.5
O1—C3—H3B	109.8	C15—C16—H16	120.5
C2—C3—H3B	109.8	O3—C17—C2	108.23 (13)
H3A—C3—H3B	108.2	O3—C17—H17A	110.1
O1—C4—C5	115.30 (13)	C2—C17—H17A	110.1
O1—C4—C9	124.24 (15)	O3—C17—H17B	110.1
C5—C4—C9	120.46 (14)	C2—C17—H17B	110.1
C6—C5—C4	119.95 (15)	H17A—C17—H17B	108.4
C6—C5—H5	120.0	O3—C18—C19	115.40 (16)
C4—C5—H5	120.0	O3—C18—C23	124.55 (15)
C7—C6—C5	120.12 (17)	C19—C18—C23	120.05 (16)
C7—C6—H6	119.9	C20—C19—C18	119.78 (19)
C5—C6—H6	119.9	C20—C19—H19	120.1
C8—C7—C6	119.77 (16)	C18—C19—H19	120.1
C8—C7—H7	120.1	C21—C20—C19	120.70 (19)
C6—C7—H7	120.1	C21—C20—H20	119.7
C7—C8—C9	121.14 (17)	C19—C20—H20	119.7
C7—C8—H8	119.4	C22—C21—C20	119.31 (19)
C9—C8—H8	119.4	C22—C21—H21	120.3
C4—C9—C8	118.54 (17)	C20—C21—H21	120.3
C4—C9—H9	120.7	C21—C22—C23	121.0 (2)
C8—C9—H9	120.7	C21—C22—H22	119.5
O2—C10—C2	108.21 (12)	C23—C22—H22	119.5
O2—C10—H10A	110.1	C18—C23—C22	119.18 (18)
C2—C10—H10A	110.1	C18—C23—H23	120.4
O2—C10—H10B	110.1	C22—C23—H23	120.4
C2—C10—H10B	110.1		

### Hydrogen-bond geometry (Å, º)

**Table:**

*D*—H···*A*	*D*—H	H···*A*	*D*···*A*	*D*—H···*A*
C5—H5···O2^i^	0.93	2.59	3.5081 (19)	170

Symmetry code: (i) *x*, *y*+1, *z*.
